# *KAT6A* amplifications are associated with shorter progression-free survival and overall survival in patients with endometrial serous carcinoma

**DOI:** 10.1371/journal.pone.0238477

**Published:** 2020-09-02

**Authors:** Ozlen Saglam, Zhenya Tang, Guilin Tang, L. Jeffrey Medeiros, Gokce A. Toruner

**Affiliations:** 1 Department of Surgical Pathology, Moffitt Cancer Center, Tampa, Florida, United States of America; 2 Department of Hematopathology, Section of Clinical Cytogenetics, The University of Texas MD Anderson Cancer Center, Houston, Texas, United States of America; CIEMAT, SPAIN

## Abstract

Somatic copy number alterations (CNA) are common in endometrial serous carcinoma (ESC). We used the Tumor Cancer Genome Atlas Pan Cancer dataset (TCGA Pan Can) to explore the impact of somatic CNA and gene expression levels (mRNA) of cancer-related genes in ESC. Results were correlated with clinico-pathologic parameters such as age of onset, disease stage, progression-free survival (PFS) and overall survival (OS) (n = 108). 1,449 genes with recurrent somatic CNA were identified, observed in 10% or more tumor samples. Somatic CNA and mRNA expression levels were highly correlated (r> = 0.6) for 383 genes. Among these, 45 genes were classified in the Tier 1 category of Cancer Genome Census-Catalogue of Somatic Mutations in Cancer. Eighteen of 45 Tier 1 genes had highly correlated somatic CNA and mRNA expression levels including *ARNT*, *PIK3CA*, *TBLXR1*, *ASXL1*, *EIF4A2*, *HOOK3*, *IKBKB*, *KAT6A*, *TCEA1*, *KAT6B*, *ERBB2*, *BRD4*, *KEAP1*, *PRKACA*, *DNM2*, *SMARCA4*, *AKT2*, *SS18L1*. Our results are in agreement with previously reported somatic CNA for *ERBB2*, *BRD4* and *PIK3C* in ESC. In addition, *AKT2* (p = 0.002) and *KAT*6A (p = 0.015) amplifications were more frequent in tumor samples from younger patients (<60), and *CEBPA* (p = 0.028) and *MYC* (p = 0.023) amplifications were more common with advanced (stage III and IV) disease stage. Patients with tumors carrying *KAT6A* and *MYC* amplifications had shorter PFS and OS. The hazard ratio (HR) of *KAT6A* was 2.82 [95 CI 1.12–7.07] for PFS and 3.87 [95 CI 1.28–11.68] for OS. The HR of *MYC* was 2.25 [95 CI 1.05–4.81] and 2.62[95 CI 1.07–6.41] for PFS and OS, respectively.

## Introduction

Somatic copy number alterations (CNA), including aneuploidy, segmental duplications and focal aberrations are frequently observed in neoplasia. For critical oncogenes and tumor suppressor genes, changes in gene copy number might result in alteration of gene expression and drive the neoplastic process. For example, *PTEN* [1] and *RB1* [2] deletions result in decreased gene expression of tumor suppressor genes, whereas *MET* [3], *ERBB2* [4] and *MYC* [5] amplifications lead to increased gene expression levels. The frequency of somatic CNAs varies significantly according to the histologic type of neoplasm as well as anatomical site. For example somatic CNAs are very common in endometrial serous cancers (ESC), but not in other endometrial cancer (EC) histologic types. As a matter of fact, ESC overlap with the “copy number (CN) high group” at the molecular level to such an extent; in the current molecular classification of EC the CN-high group is also known “serous-like” carcinoma [6]. ESC is one of the high-grade EC with a worse clinical outcome compared to low-grade (type 1) EC [7].

We hypothesized that frequently observed somatic CNAs are highly relevant in the pathogenesis of ESC by changing expression levels of critical cancer-related genes. To address this hypothesis, we took advantage of the publically available TCGA Pan Can dataset deposited in cBioPortal (cbioportal.org). The main objective of this study is to identify candidate oncogenes and tumor suppressor genes, which have been implicated in other human neoplasms, but not implicated ESC. We pursued this objective by correlating copy number and mRNA expression levels in patients with ESC using the TCGA Pan Cancer dataset (TCGA Pan Can), and cross-tabulating the highly correlated genes with known cancer genes (i.e. Tier 1 Cancer Census Genes for Catalogue of Somatic Mutations in Cancer (CGC-COSMIC) [8]. The secondary objective of this study is to explore associations of identified Tier 1 CGC-COSMIC genes with clinic-pathological parameters such as disease stage, age of onset, overall survival (OS) and progression free survival (PFS), to identify potential biomarkers associated with these cancers.

## Material and methods

### TCGA endometrial serous cancer cohort

ESC samples (n = 108) were identified in public TCGA dataset [9] from cBioPortal [10]. Frozen tumor samples with companion normal tissue were collected at diagnosis according to the consent provided by the relevant institutional review boards of participating institutions. Patients were selected only if their treatment plan required surgical resection and had received no prior treatment for their disease. Pathologic diagnoses were made at local laboratories using formalin-fixed and paraffin-embedded (FFPE) sections. Each frozen, OCT-embedded tumor was processed centrally by the TCGA and a hematoxylin-eosin stained section was reviewed by a pathologist to confirm the tumor subtype and grade [6, 9]. For a given patient, clinical data such as age of onset, stage of tumor, OS and PFS were extracted using the visualization tools of cBioPortal.

### Identification of recurrent somatic CNA

Copy number status of each gene (n = 24,881) in the genome was determined according to the TCGA analysis methods described elsewhere. Using cBioPortal tools [10], the dataset specifying somatic copy number aberration (CNA) frequency for each gene in 108 patients was downloaded. Then, 1,449 genes with recurrent somatic CNA (i.e. amplification or deletion in at least 10% of tumor samples) were identified (S1 Table). GRCh38 coordinates of each identified gene was obtained from the Galaxy platform [11] (S2 Table). Based on these coordinates, genomic blocks with recurrent somatic CNAs were determined (Table 1).

**Table 1:** Recurrent somatic copy number aberrations (CNAs) in endometrial serous carcinoma.

Locus	Cytoband	GRC38 Coordinates	Size (Mb)	CNV	Tier 1 COSMIC-CGC genes
1	1p34.3-p34.2	chr1:38991243–43623672	4.63	AMP	*MYCL*
2	1p12-p11.2	chr1:119911552–121571888	1.66	AMP	
3	1q21.1-q22	chr1:143874742–156429548	12.55	AMP	*BCL9*,*MLLT11*,***ARNT***^a^,*TPM3*, *LMNA*,*MUC1*
4	2q14.1-q14.1	chr2:112773914–113756693	0.98	AMP	*PAX8*
5	3q26.1-q29	chr3:165772902–198222513	32.45	AMP	*MECOM*,***PIK3CA***^a^,***TBL1XR1***^a^,*SOX2*, *MAP3K13*, *BCL6*, ***EIF4A2***, *LPP*, *TP63*
6	5p15.33-p15.33	chr5:1392789–2312201	0.92	AMP	
7	5p15.31-p15.2	chr5:9035025–9903824	0.87	AMP	*LIFR*, *IL7R*
8	5p13.3-p13.3	chr5:31639409–32791724	1.15	AMP	
9	5p13.2-p13.1	chr5:35617886–41870689	6.25	AMP	
10	8p23.3-p23.2	chr8:208343–4994806	4.79	DEL	
11	8p21.3-p21.1	chr8:22275279–27992852	5.72	DEL	
12	8p21.1	chr8:41261956–43363185	2.10	AMP	***HOOK3***^a^**,*IKBKB***^a^**,*KAT6A***^a^
13	8q12.1-q12.1	chr8:53851807–70404238	0.78	AMP	*NCOA2***, *TCEA1***^a^
14	8q22.1-q22.3	chr8:94371959–101669726	7.30	AMP	
15	8q24.13-q24.31	chr8:124310917–141228574	16.92	AMP	*MYC*, *NDRG1*
16	10q22.2-q22.3	chr10:74151184–79067448	4.92	AMP	***KAT6B***^a^
17	11q13.1-q13.2	chr11:63838927–67410090	3.57	AMP	
18	17q12-q21.1	chr17:39461510–40136789	0.68	AMP	***ERBB2***^a^**,** *CDK12*
19	17q25.1-q25.1	chr17:75318075–75825805	0.51	AMP	
20	18p11.31-p11.3	chr18:3066806–4455266	1.39	AMP	
21	18q11.2-q11.2	chr18:26226882–26657512	0.43	AMP	
22	19p13.2-p13.11	chr19:8809575–18323191	9.51	AMP	*JAK3*,***BRD4***^a^,*DNAJB1*,***PRKACA***^a^,*TPM4*,*LYL1*,*CALR*,***DNM2***^a^,***KEAP1***^a^, ***SMARCA4***, ^a^
23	19q11-q13.2	chr19:27790492–40465818	12.68	AMP	*CEBPA*,*CCNE1*,***AKT2***
24	20q11.21-q11.23	chr20:31257663–37241623	5.98	AMP	***ASXL***^a^***1***
25	20q13.12-q13.2	chr20:44966469–53583097	8.62	AMP	*SDC4*
26	20q13.33-q13.33	chr20:62143719–62762771	0.62	AMP	**SS18L1**^a^

^a^The genes in bold format have correlation coefficient—r >0.6

### Correlation of gene expression and copy number data for genes with recurrent somatic CNA

Relative linear copy number values were plotted against mRNA expression z-scores (RNA Seq V2) in order to determine the impact of somatic CNA on gene expression at the mRNA level, Pearson correlation coefficients were obtained using the cBioPortal visualization tool. The cut-off for “high-correlation” was arbitrarily accepted as equal as or more than 0.6 (r > = 0.6).

### Identification of cancer relevant genes among genes with recurrent somatic CNAs

In order to identify cancer relevant genes among the genes with recurrent somatic CNA, we cross-tabulated these genes with Tier 1 Cancer Gene Census (CGC) genes (n = 576) from the Catalogue of Somatic Mutations in Cancer (COSMIC) (S3 Table). CGC is an ongoing curation effort under the auspices of COSMIC to catalogue genes whose mutations have been causally implicated in cancer. To be classified into Tier 1, a gene must possess a documented activity relevant to cancer, along with evidence of mutations in cancer which change the activity of the gene product in a way that promotes oncogenic transformation [8]. From cBioPortal, somatic CNA and point mutation data were obtained.

### Association of cancer relevant genes with clinic-pathological data

GraphPad Prism (v8.0.0) and Minitab software (v18) were used for statistical analysis. Fisher exact tests were applied for categorical variables such as somatic CNA, stage of disease and age of diagnosis. For age, we used 60 years as an arbitrary cut-off since ESC is usually diagnosed at more advanced ages, typically in the eight decade [12]. The Kaplan-Meier method was used to estimate PFS and OS at last follow-up date for alive patients with no evidence of progression for PFS estimation. For OS, just being alive is qualified for censoring. Using the curve comparison analysis module in GraphPad Prism software (v8.0.0), the median PFS differences, hazard ratios (Mantel-Haentzel) and p-values (Mantel-Cox test) were calculated. *p* <0.05 was considered to be statistically significant.

## Results

### Cohort characteristics

The median patient age was 68 years (range, 45–90). There were 38 patients with stage I, 12 with stage II, 45 with stage III and 13 with stage IV disease. The patients < 60 years of age presented more often with advanced stage (III and IV) disease compared to older patients (p = 0.002). More advanced disease stage was associated with shorter PFS (p<0.001) and OS (p<0.001).

### Recurrent somatic CNA in endometrial serous carcinoma

1,449 genes with somatic CNA were observed in at least 10% of tumors (S1 Table) and were located on 26 genomic segments on chromosomes 1, 2, 3, 5, 8, 10, 11, 17, 18, 19 and 20. The size of these genomic loci ranged from 0.43 to 32.45 MB (median, 4.10). With the exception of two segments exhibiting deletion on 8p, spanning 8p23.3 to 8p21.1 (chr8:208343–27992852), all detected somatic CNAs were amplifications (Table 1).

### Impact of recurrent somatic CNA in endometrial serous carcinoma

**Fig 1:**
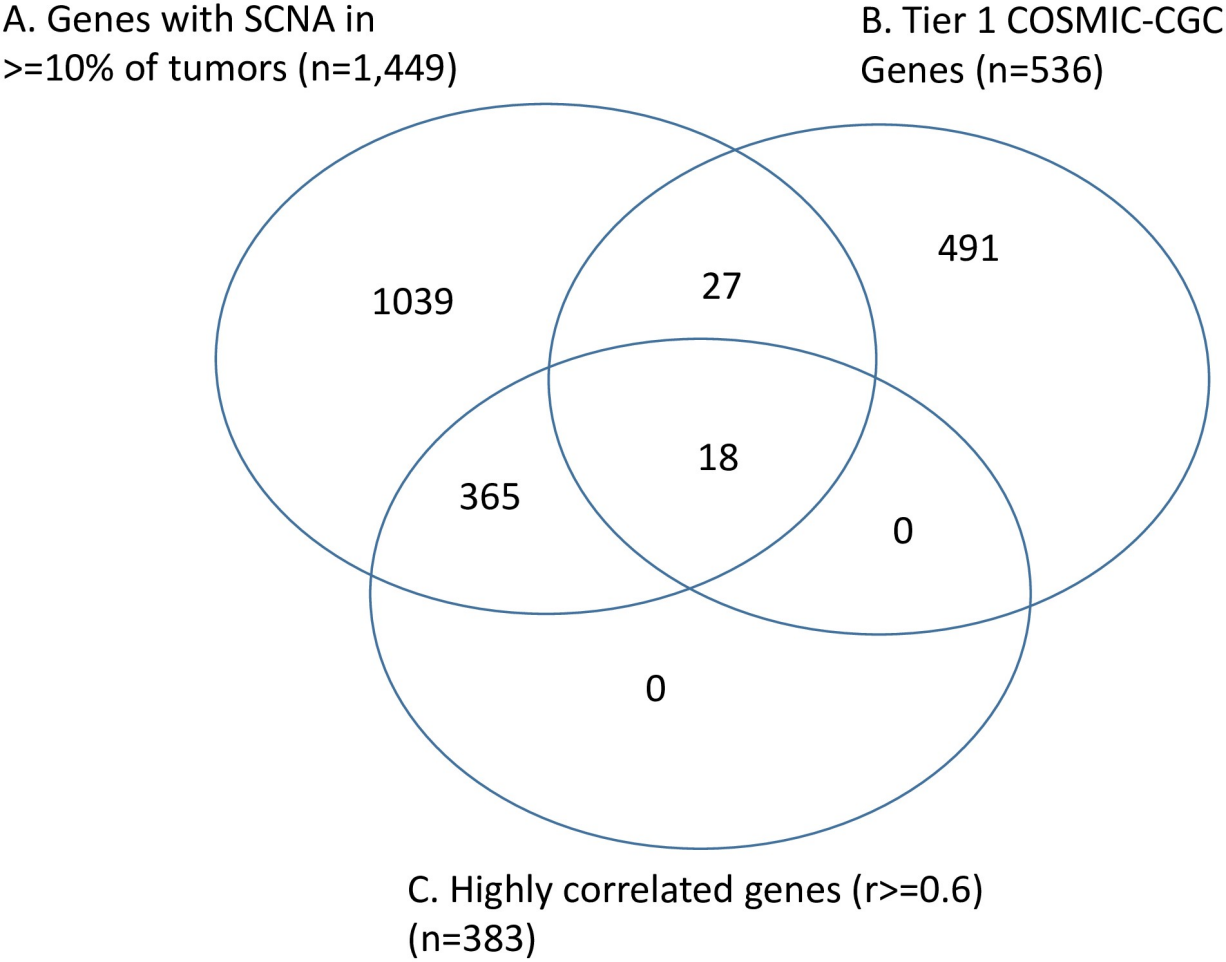
Venn diagram of genes with somatic CNA in > = 10 of tumors, Tier 1 CGC-COSMIC genes and highly correlated genes.

The 1,449 genes with recurrent somatic CNA were evaluated based on two criteria. The first criterion was whether the gene copy number and the gene expression was highly correlated (r> = 0.6), and the second criterion was whether the gene was implicated in cancer (Tier 1 CGC-COSMIC gene). The number of the genes that fulfilled the first (highly correlated) and second (implicated in cancer) criteria were 383 and 45, respectively. In addition, 18 genes fulfilled both criteria, as they are highly correlated Tier 1 CGC-COSMIC genes (Fig 1).

**Fig 2:**
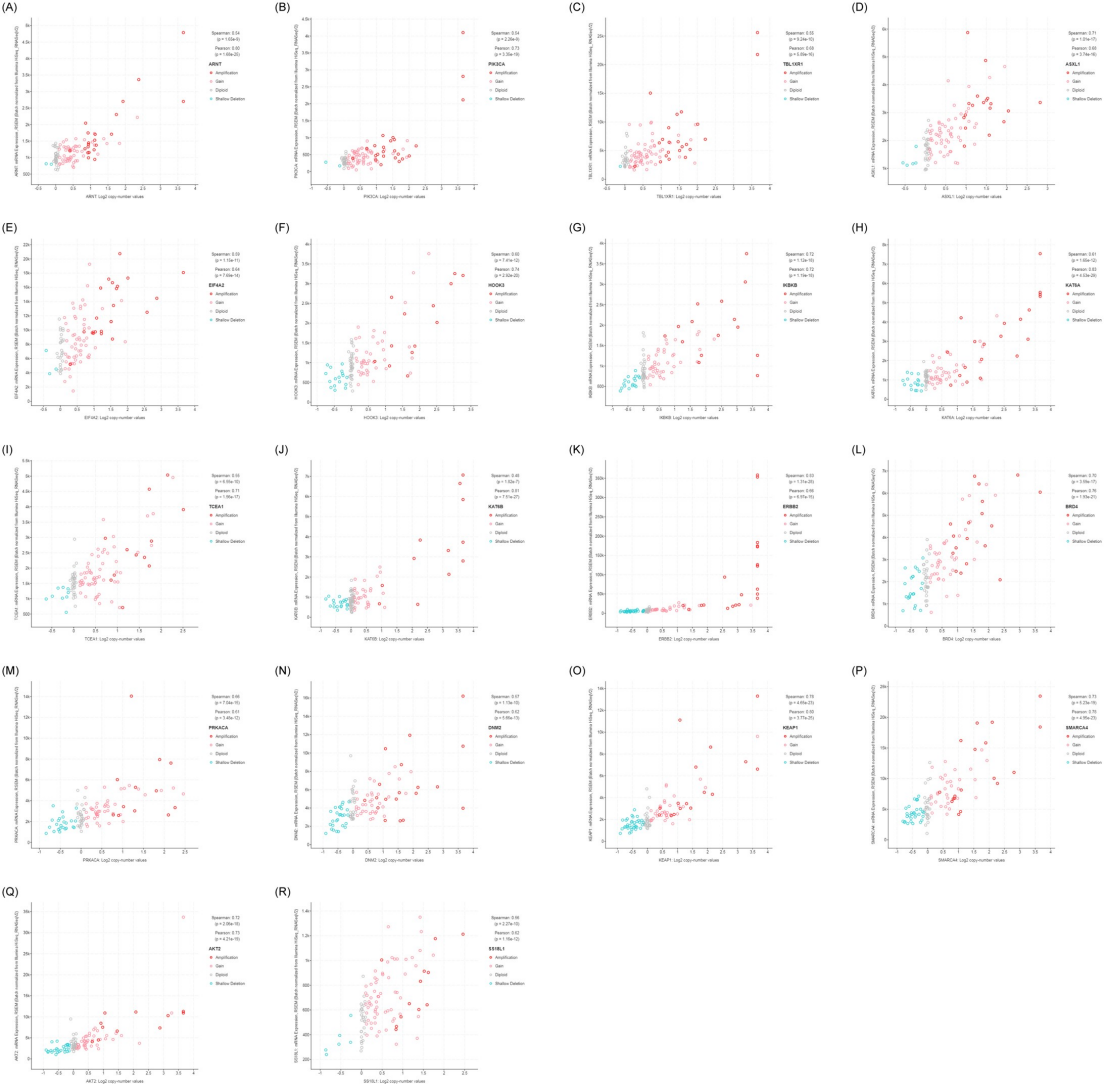
Correlation of gene copy numbers with mRNA expression. A. *ARNT*, B.*PIK3CA*, C. *TBL1XR1*, D.*ASXL1*, E.*EIF4A2*, F.*HOOK3*, G.*IKBKB*, H. *KAT6A*, I.*TCEA1*, J.*KAT6B*, K. *ERBB2*, L.*BRD4*, M.*PRKACA*, N.*DNM2*, O.*KEAP1*, P.*SMARCA4*, Q.*AKT2*, R.*SS18L1*.

These 18 genes were *ARNT* (r = 0.80), *PIK3CA* (r = 0.73), *TBL1XR1*(r = 0.68), *ASXL1* (r = 0.68), *EIF4A2* (r = 0.64), *HOOK3* (r = 0.74), *IKBKB* (r = 0.72), *KAT6A* (r = 0.83), *TCEA1*(r = 0.71), *KAT6B* (r = 0.81), *ERBB2* (r = 0.66), *BRD4* (r = 0.76), *PRKACA* (r = 0.62), *DNM2*(r = 0.62), *KEAP1*(r = 0.8), *SMARCA4* (r = 0.78), *AKT2* (r = 0.73), *SS18L1* (r = 0.62) (Fig 2A–2Q).

Eleven of these genes were located on the same genomic segment, such as *PIK3CA*, *TBLIXR1* and *EIF4A2* at 3q26.1-q29; *HOOK3*, *IKBKB* and *KAT6A* at 8p21.1; and *BRD4*, *PRKACA*, *DNMA2*, *KEAP1* and *SMARCA4* 19p13.2-p13.11 and they were co-amplified (Table 1; S4 Table).

### Mutation spectrum of Tier 1 CGC-COSMIC genes

**Fig 3:**
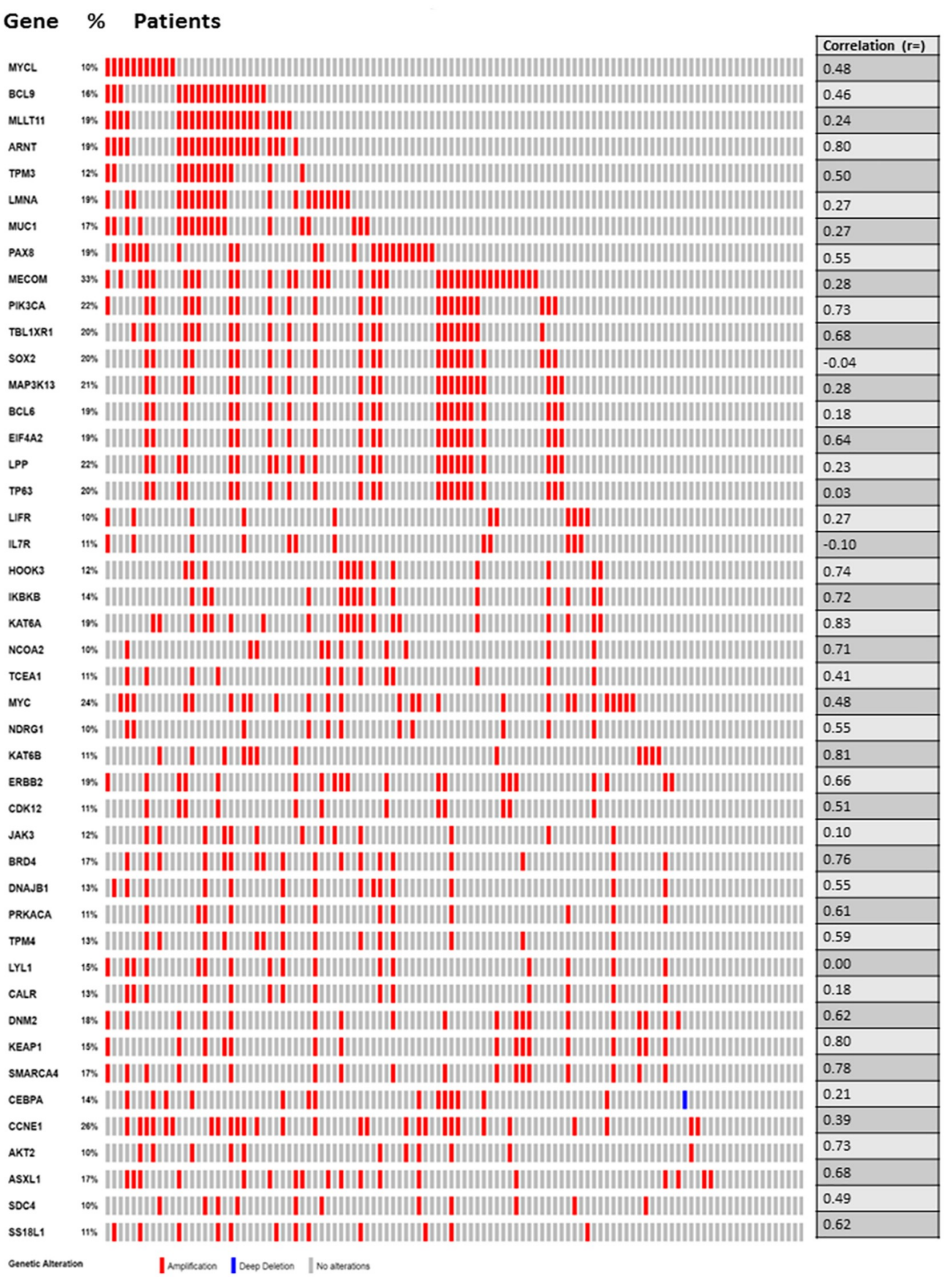
Somatic CNA of 45 Tier 1 CGC-COSMIC Genes occurring in the endometrial serous cancers in the TCGA PanCan dataset (n = 108).

The most observed aberrations were amplifications (Fig 3) with the mutation rate ranging 11 to 33% of the tumors. Point mutation or fusions were infrequently observed in these tumors with a notable exception of *PIK3CA*. *PIK3CA* was mutated in 37% of tumor samples (S1A Fig, and when combined with amplifications, 51% of samples had a *PIK3CA* abnormality (S1B Fig). A large majority of these *PIK3CA* mutations were characterized as Oncogenic/Likely Oncogenic mutations in OncoKB. Among other genes, rate of point mutations varied between 0.9 to 5%. For 45 genes, fusions involving *PAX8* (n = 1), *MUC 1* (n = 1), *MECOM* (n = 2), *MAP3K13* (n = 1), *ERBB2* (n = 2), *DNM2* (n = 1), *KEAP1* (n = 1), *SMARCA4* (n = 1), and *AKT2* (n = 1) were found in 11 out of 108 tumors (~11%) (S1A Fig).

### Association of Tier 1 CGC-COSMIC gene amplifications with clinico-pathological parameters

#### Association with age

The frequency of *AKT2 and KAT*6A amplifications were much higher in patients who were younger than 60 years. For *AKT2*, the frequency of amplifications for younger patients was 36% (n = 11), whereas this figure was 7% for older patients (n = 97) (p = 0.002). For *KAT6A*, younger and older patients had amplification rates of 45% and 15%, respectively (p = 0.015). There was no association between age and other genes (Table 2).

**Table 2:** Summary data on association and correlation for Tier 1 CCG-COSMIC genes.

Genomic Segment	Tier 1 CGC-COSMIC genes	Correlation with RNA expression (r=)	Association with Stage	Association with Age	Association with PFS or OS
1p34.3-p34.2	*MYCL*	0.48	NS	NS	NS
1q21.1-q22	*BCL9*	0.46	NS	NS	NS
1q21.1-q22	*MLLT11*	0.24	NS	NS	NS
1q21.1-q22	*ARNT*	**0.80**	NS	NS	NS
1q21.1-q22	*TPM3*	0.50	NS	NS	NS
1q21.1-q22	*LMNA*	0.27	NS	NS	NS
1q21.1-q22	*MUC1*	0.27	NS	NS	NS
2q14.1-q14	*PAX8*	0.55	NS	NS	NS
3q26.1-q29	*MECOM*	0.28	NS	NS	NS
3q26.1-q29	*PIK3CA*	**0.73**	NS	NS	NS
3q26.1-q29	*TBL1XR1*	**0.68**	NS	NS	NS
3q26.1-q29	*SOX2*	-0.04	NS	NS	NS
3q26.1-q29	*MAP3K13*	0.28	NS	NS	NS
3q26.1-q29	*BCL6*	0.18	NS	NS	NS
3q26.1-q29	*EIF4A2*	**0.64**	NS	NS	NS
3q26.1-q29	*LPP*	0.23	NS	NS	NS
3q26.1-q29	*TP63*	0.03	NS	NS	NS
3q26.1-q29	*LIFR*	0.27	NS	NS	NS
3q26.1-q29	*IL7R*	-0.10	NS	NS	NS
8p21.3-p21.1	*HOOK3*	**0.74**	NS	NS	NS
8p21.3-p21.1	*IBKBB*	**0.72**	NS	NS	NS
8p21.3-p21.1	*KAT6A*	**0.83**	NS	**p = 0.015**	**PFS (p = 0.027); OS (p = 0.016)**
8q12.1-q12.1	*TCEA1*	**0.71**	NS	NS	NS
8q12.1-q12.1	*NCOA2*	0.41	NS	NS	NS
8q24.13-q24	*MYC*	0.48	**p = 0.023**	NS	**PFS (p = 0.035); OS (p = 0.033**)
8q24.13-q24	*NDRG1*	0.55	NS	NS	NS
10q22.2-q22	*KAT6B*	**0.81**	NS	NS	NS
17q12-q21.1	*ERBB2*	**0.66**	NS	NS	NS
17q12-q21.1	*CDK12*	0.51	NS	NS	NS
19p13.2-p13.11	*JAK3*	0.10	NS	NS	NS
19p13.2-p13.11	*BRD4*	**0.76**	NS	NS	NS
19p13.2-p13.11	*DNJAB1*	0.55	NS	NS	NS
19p13.2-p13.11	*PRKACA*	**0.61**	NS	NS	NS
19p13.2-p13.11	*TPM4*	0.59	NS	NS	NS
19p13.2-p13.11	*LYL1*	0.00	NS	NS	NS
19p13.2-p13.11	*CALR*	0.18	NS	NS	NS
19p13.2-p13.11	*DNM2*	0.62	NS	NS	NS
19p13.2-p13.11	*KEAP1*	**0.80**	NS	NS	NS
19p13.2-p13.11	*SMARCA4*	**0.78**	NS	NS	NS
19q11-q13.2	*CEBPA*	0.21	**p = 0.028**	NS	NS
19q11-q13.3	*CCNE1*	0.39	NS	NS	NS
19q11-q13.2	*AKT2*	0.73	NS	**p = 0.003**	NS
20q11.21-q11.23	*ASXL1*	0.68	NS	NS	NS
20q13.12-q13.2	*SDC4*	0.49	NS	NS	NS
20q13.33-q13.33	*SS18L1*	0.62	NS	NS	NS

#### Association with disease stage

The frequency of *CEBPA and MYC* amplifications was much higher in patients with advanced stage disease. For *CEBPA*, the frequency of amplifications in patients diagnosed at advanced stage (n = 58) was 21%, whereas it was 6% for patients diagnosed at stage I and II disease (n = 50) (p = 0.028). For *MYC*, tumor samples obtained from advanced and early stage diseases had an amplification rate of 33% and 14%, respectively (p = 0.023). An association with other genes was not observed (Table 2).

#### Association with PFS and OS

**Fig 4:**
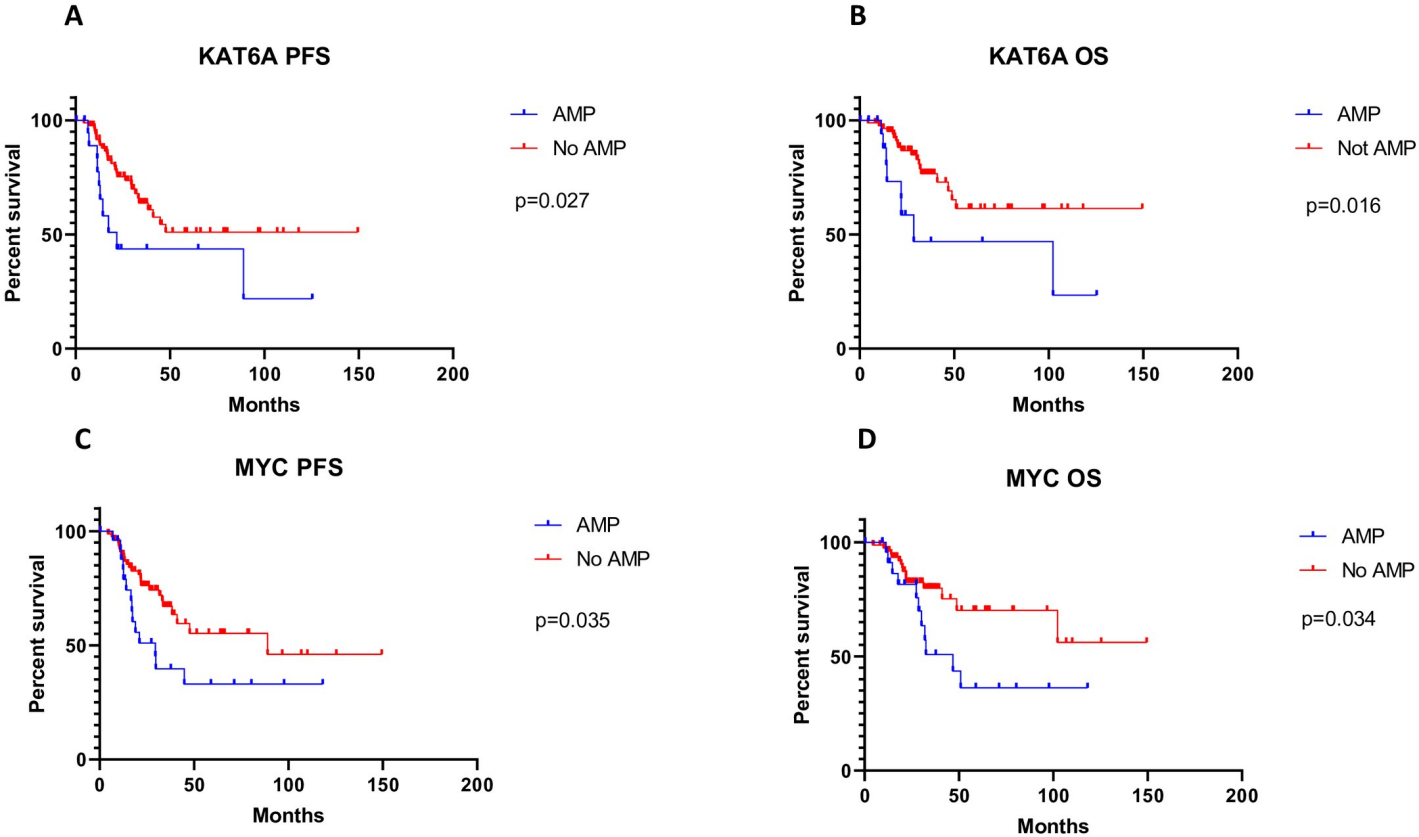
Comparison of survival curves A. *KAT6A* progression-free survival Amp versus. No-AMP; B. *KAT6A* overall survival Amp versus. no-AMP; C. *MYC* progression-free survival Amp versus. No-AMP; D. *MYC* overall survival Amp vs. no-AMP.

Patients with tumors carrying *KAT6A* and *MYC* amplifications had shorter PFS and OS. For *KAT6A*, the hazard ratio (HR) for PFS is 2.82 [95 CI 1.12–7.07] (p = 0.027), and the HR for OS was 3.87 [95 CI 1.28–11.68] (p = 0.016) (Fig 4A and 4B). For *MYC*, the HR for PFS was 2.25 [95 CI 1.05–4.81] (p = 0.035), and the HR for OS was 2.62[95 CI 1.07–6.41] (p = 0.034) (Fig 4C and 4D).

## Discussion

The primary objective of this study was to identify candidate oncogenes and tumor suppressor genes in ESC cases by correlating DNA copy number and mRNA expression in the TCGA cohort. We identified 18 amplified known oncogenes, which also were overexpressed in ESC. Our PubMed search of these 18 genes in relation to “endometrial cancer”, “endometrial serous cancer” and “gynecologic cancers” categorized them into four groups. The first group of genes were reported previously in ESC pathogenesis. For *ERBB2* [13], *BRD4* [14] and *PIK3CA* [15], our findings are in accord with previously reported findings on ESC suggesting overexpression due to amplification. The second group of genes has been implicated in EC, but not in the serous histologic type. These include *IKBKB* [16, 17], *KEAP1* [18, 19], *AKT2* [20, 21] and *SMARCA4* [22]. The third group of genes are *ARNT* [23], *KAT6B* [24], *DNM2* [25] and *ASXL1* [26]. These genes have been reported to be associated with other gynecological cancers, but not in endometrial cancers. To the best of our knowledge, *TBL1XR1*, *EIF4A2*, *HOOK3*, *KAT6A*, *TCEA1*, *PRKACA*, and *SS18L1* belong to a group of genes that have never been implicated in any gynecological cancers. Point mutation or fusions of the amplified genes were not frequently observed in these tumors, with a notable exception of *PIK3CA*. Point mutations and amplifications of *PIK3CA* were observed in approximately half of the tumor samples.

The secondary objective of this study was to explore potential associations of identified cancer genes with clinic-pathological parameters. For this purpose, association of several genes at three recurrent somatic CNA, at 8p21.3, 8q24.13 and 19q11-q13.2, is worth mentioning. The 2.1 Mb recurrent somatic CNA at 8p21.1 (chr8:41261956–43363185) contains three Tier 1 CGC-COSMIC genes *HOOK3*, *IBKB* and [25] *KAT6A*. Expression of all three genes was highly correlated with their copy numbers. No associations with clinic-pathologic parameters were noted for *HOOK3* or *IKBKB*, but KAT6A amplification was associated with shorter PFS and OS and earlier age of onset of disease. *KAT6A* has never been implicated in gynecological malignancies, and its role is unknown in ESC. *KAT6A* is a member of the histone lysine acetyltransferase (KATs) family, also known as monocytic leukemia zinc finger protein (MOZ). *KAT6A* has an important role in the regulation of chromatin organization and function. Translocations involving *KAT6A* (and *KAT6B)* is are identified in acute myeloid leukemia [27]. In an animal study, inhibitors of *KAT6A/B* induced senescence and arrest in lymphoma growth [28]. Even partial blockage of *KAT6A* reduced proliferation of myc-induced lymphoma and leukemia [29]. Our results indicate *KAT6A* is one of the candidate genes for further evaluation in ESC pathogenesis.

**Fig 5:**
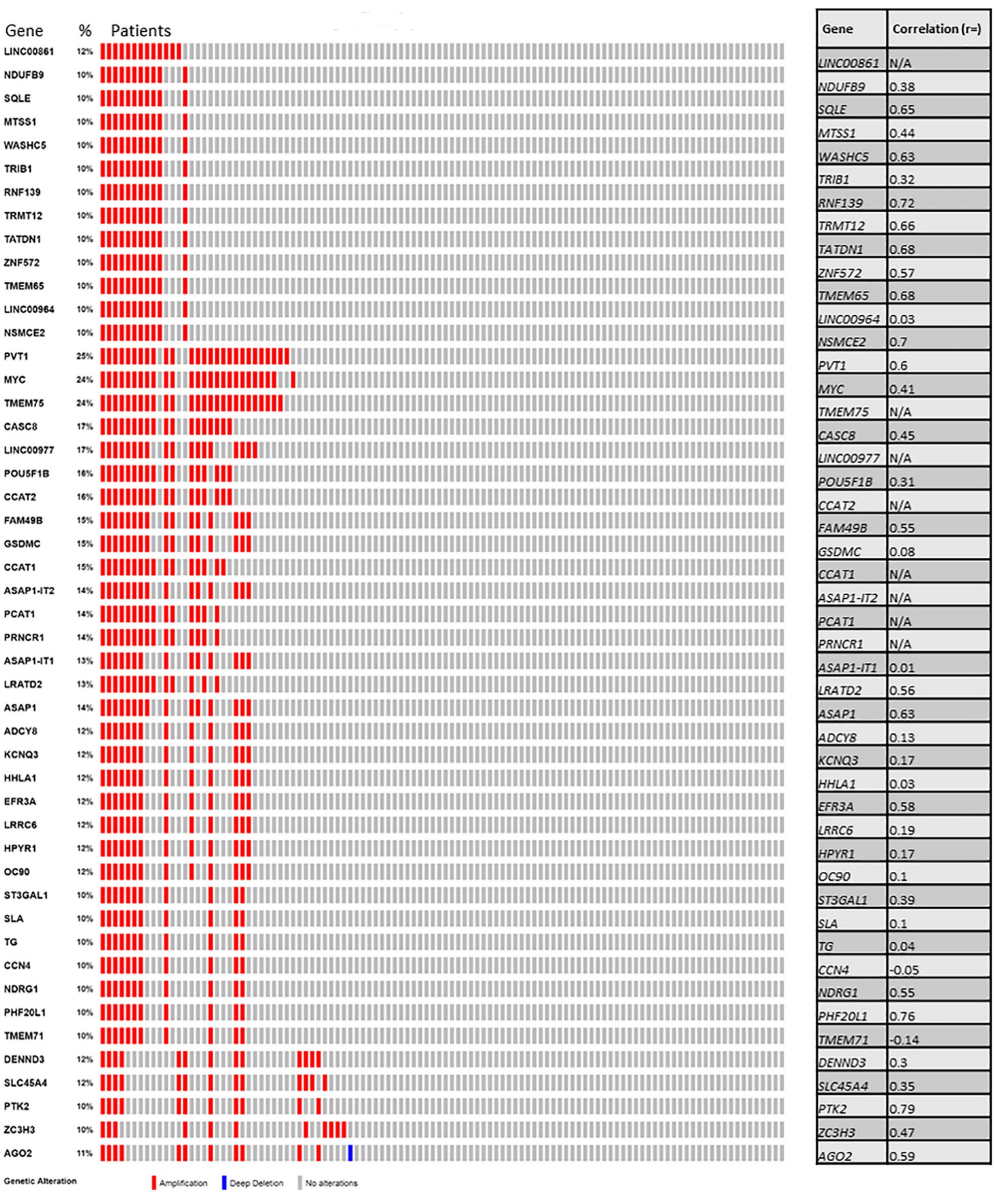
Somatic CNA of 47 genes in the 16.92 MB recurrent somatic CNA genomic segment chr8:124310917–141228574 at 8q24.13-q24.3 occurring in the endometrial serous cancers of the TCGA PanCan dataset (n = 108).

The 16.92 MB recurrent somatic CNA at 8q24.13-q24.31 (chr8:41261956–43363185) contains two Tier 1 CGC-COSMIC genes, *MYC and NDRG1*. Neither *MYC (r = 0*.*48)* nor *NDRG1* (r = 0.55) expression was highly correlated with their copy numbers. However, amplification of *MYC* was associated with higher disease stage and poorer OS and PFS rates. *MYC* amplification in EC has been reported in other studies [30, 31]. In agreement with our results, *MYC* amplification along with HER-2/neu and cyclin E high protein expression have been associated with tumor progression, higher tumor grade and deep myometrial invasion in the literature [32]. Although *MYC* copy number and mRNA expression levels were not highly correlated, *PVT1* was co-amplified with *MYC* (p<0.001) (Fig 5) in 25% of the cases, and had a strong correlation with gene expression (r = 0.60). *PVT*1 is not a CGC-COSMIC gene. but it encodes a long non-coding RNA with oncogenic properties whose amplification and overexpression have been implicated in several cancers including breast and ovarian carcinomas [33]. Therefore, *PVT1*, in conjunction with or instead of *MYC* might be the cancer driver gene in this setting.

The 12.16 MB recurrent somatic CNA at 19q11-13.2 (chr8:41261956–43363185) contains three Tier 1 CGC-COSMIC genes *CEBPA*, *CCNE1*, *AKT2*. Expression of *AKT2* was highly correlated with its copy number (r = 0.73), whereas this correlation was weaker for *CEBPA* (r = 0.23) and *CCNE*1(r = 0.39). AKT2 amplification was associated with younger age of disease onset.*AKT2* belongs to a family of three serine/threonine-protein kinases called the AKT kinases (encoded by *AKT1*, *AKT2* and *AKT3*), which regulate cell proliferation, cell survival, growth and angiogenesis. Among all three genes, amplification and overexpression of *AKT2* was demonstrated in many cancers including EC.AKT2 was associated with cancer cell invasion, metastasis, and survival [34]. A second amplified gene at the same locus was *CEBP* in our analysis. Its amplification was also associated with more advanced disease stage. *CEBPA* expression is highly expressed in normal endometrial tissues and is not expressed in clinical endometrial cancer samples [35]. DNA hypermethylation of the upstream *CEBPA* promoter region is responsible for very low *CEBPA* expression in lung and endometrial cancers [36]. Decreased expression of *CEBPA* by posttranscriptional regulation was also shown in myeloid leukemia [32]. Therefore, CEBPA does not appear to be a likely candidate driver gene in ESC. Lastly, *CCNE1* amplification was one of the previously reported [37–39] genes in EC, and we also identified *CCNE1* amplification in serous-like cancers of TGCA samples. However, we did not detect a strong correlation (r = 0.39) between copy number and mRNA expression levels for this gene. It is plausible that *AKT2* rather than *CCNE1 or CEBPA* in this dataset is the driver of the tumor. More detailed studies are required for this group of genes at this locus.

There are several limitations of this study. First, this is a retrospective analysis of a multi-center study and the obtained survival data in regard to PFS and OS are not derived from a randomized clinical trial, or even the clinical practice of a single center. Therefore some heterogeneity is expected. A second limitation is regarding the calls for somatic CNA. GISTIC [40] is the standard algorithm to call somatic CNA in the TCGA studies using Affymetrix data, however other algorithms like Hidden Markov Models(HMM) [41] and Circular Binary Segmentation (CBS) [42] are widely used in commercial software applied in clinical practice. For the purpose of this study, we accepted TCGA calls at their face value. A third limitation of is the arbitrary selection of a cut-off for Pearson coefficient (r = > = 0.6). However, this approach seems adequately sensitive to identify *ERBB2* [13], *BRD4* [14] and *PIK3CA* [15], in agreement with previously generated data. A fourth limitation is using CCG-COSMIC Tier 1 genes as the source for “cancer genes”. There are other initiatives for curation of cancer genes, such as CIViC [43], myCancerGenome [44] or OnkoKB [45], but we believe CGC-COSMIC is a reputable dataset. A fifth limitation is using mRNA levels as a marker of gene expression. Obviously, protein levels from reverse phase protein arrays (RPPA) and immunohistochemistry studies would be ideal, but these data are not available for EC in the TCGA dataset. Finally, the major limitation of this study is the analysis being performed only on the TCGA dataset. Although ESC collection (n = 108) in the TCGA Pan Cancer dataset is the largest known cohort with clinical and genomics data on these tumors, we are aware that in an ideal situation, our findings should be replicated in an independent study group. Unfortunately, the rarity of the ESC, precluded the prospective collection of same of higher number of tumors with relevant clinical information for the purposes of this study.

In conclusion, despite the aforementioned limitations, our analysis of ESC in TCGA samples identified several novel candidate genes which may be important in the ESC pathogenesis. *KAT6A* is among the most interesting and novel candidate; amplification correlated with increased gene expression and was associated with low PFS and OS. The results of this study also reaffirm the previously known clinic-pathological associations for loci such as 8q24and 19q13-q11. More research is warranted to determine the impact of gene copy number changes in the pathogenesis of ESC.

## Supporting information

S1 Table Genes with recurrent somatic CNA in at least 10% of tumors. (XLSX)

S2 Table GRCh38 coordinates of identified genes. (XLSX)

S3 Table Tier 1 Cancer Gene Census (CGC) genes from the Catalogue of Somatic Mutations in Cancer (COSMIC). (XLSX)

S4 Table Co-occurrence and mutual exclusivity analysis of Tier 1 CGC-COSMIC genes. (XLSX)

S1 Fig A. Somatic mutations. B. Combined somatic mutations and somatic copy number alterations in 45 Tier 1 CGC-COSMIC genes occurring in the endometrial serous cancers of the TCGA PanCan dataset.
(TIF)

## References

[1] Whang YE, Wu X, Suzuki H, Reiter RE, Tran C, Vessella RL, et al Inactivation of the tumor suppressor PTEN/MMAC1 in advanced human prostate cancer through loss of expression. Proc Natl Acad Sci U S A. 1998;95(9):5246–50. Epub 1998/06/06. 10.1073/pnas.95.9.5246 . 9560261 PMC20246

[2] Zhang X, Xu HJ, Murakami Y, Sachse R, Yashima K, Hirohashi S, et al Deletions of chromosome 13q, mutations in Retinoblastoma 1, and retinoblastoma protein state in human hepatocellular carcinoma. Cancer Res. 1994;54(15):4177–82. Epub 1994/08/01. . 8033150

[3] Cooper CS, Tempest PR, Beckman MP, Heldin CH, Brookes P. Amplification and overexpression of the met gene in spontaneously transformed NIH3T3 mouse fibroblasts. EMBO J. 1986;5(10):2623–8. Epub 1986/10/01. . 3023053 10.1002/j.1460-2075.1986.tb04543.x PMC1167161

[4] Hudziak RM, Lewis GD, Shalaby MR, Eessalu TE, Aggarwal BB, Ullrich A, et al Amplified expression of the HER2/ERBB2 oncogene induces resistance to tumor necrosis factor alpha in NIH 3T3 cells. Proc Natl Acad Sci U S A. 1988;85(14):5102–6. Epub 1988/07/01. 10.1073/pnas.85.14.5102 . 2899323 PMC281696

[5] Nau MM, Carney DN, Battey J, Johnson B, Little C, Gazdar A, et al Amplification, expression and rearrangement of c-myc and N-myc oncogenes in human lung cancer. Curr Top Microbiol Immunol. 1984;113:172–7. Epub 1984/01/01. 10.1007/978-3-642-69860-6_29 . 6090062

[6] Cancer Genome Atlas Research N, Kandoth C, Schultz N, Cherniack AD, Akbani R, Liu Y, et al Integrated genomic characterization of endometrial carcinoma. Nature. 2013;497(7447):67–73. Epub 2013/05/03. 10.1038/nature12113 . 23636398 PMC3704730

[7] Murali R, Davidson B, Fadare O, Carlson JA, Crum CP, Gilks CB, et al High-grade Endometrial Carcinomas: Morphologic and Immunohistochemical Features, Diagnostic Challenges and Recommendations. Int J Gynecol Pathol. 2019;38 Suppl 1:S40–S63. Epub 2018/12/15. 10.1097/PGP.0000000000000491 . 30550483 PMC6296248

[8] Sondka Z, Bamford S, Cole CG, Ward SA, Dunham I, Forbes SA. The COSMIC Cancer Gene Census: describing genetic dysfunction across all human cancers. Nat Rev Cancer. 2018;18(11):696–705. Epub 2018/10/08. 10.1038/s41568-018-0060-1 . 30293088 PMC6450507

[9] Berger AC, Korkut A, Kanchi RS, Hegde AM, Lenoir W, Liu W, et al A Comprehensive Pan-Cancer Molecular Study of Gynecologic and Breast Cancers. Cancer Cell. 2018;33(4):690–705 e9. Epub 2018/04/07. 10.1016/j.ccell.2018.03.014 . 29622464 PMC5959730

[10] Gao J, Aksoy BA, Dogrusoz U, Dresdner G, Gross B, Sumer SO, et al Integrative analysis of complex cancer genomics and clinical profiles using the cBioPortal. Sci Signal. 2013;6(269):pl1 Epub 2013/04/04. 10.1126/scisignal.2004088 . 23550210 PMC4160307

[11] Afgan E, Baker D, Batut B, van den Beek M, Bouvier D, Cech M, et al The Galaxy platform for accessible, reproducible and collaborative biomedical analyses: 2018 update. Nucleic Acids Res. 2018;46(W1):W537–W44. Epub 2018/05/24. 10.1093/nar/gky379 . 29790989 PMC6030816

[12] Lokich E, Kole M, Raker C, Quddus MR, Mathews C. Molecular markers in uterine serous cancer: Correlation between endometrial biopsy and hysterectomy specimens. Gynecol Oncol Rep. 2019;29:98–101. Epub 2019/08/31. 10.1016/j.gore.2019.04.005 . 31467960 PMC6710608

[13] Slomovitz BM, Broaddus RR, Burke TW, Sneige N, Soliman PT, Wu W, et al Her-2/neu overexpression and amplification in uterine papillary serous carcinoma. J Clin Oncol. 2004;22(15):3126–32. Epub 2004/07/31. 10.1200/JCO.2004.11.154 . 15284264

[14] Jones DH, Lin DI. Amplification of the NSD3-BRD4-CHD8 pathway in pelvic high-grade serous carcinomas of tubo-ovarian and endometrial origin. Mol Clin Oncol. 2017;7(2):301–7. Epub 2017/08/07. 10.3892/mco.2017.1289 . 28781807 PMC5532634

[15] Holst F, Werner HMJ, Mjos S, Hoivik EA, Kusonmano K, Wik E, et al PIK3CA Amplification Associates with Aggressive Phenotype but Not Markers of AKT-MTOR Signaling in Endometrial Carcinoma. Clin Cancer Res. 2019;25(1):334–45. Epub 2018/11/18. 10.1158/1078-0432.CCR-18-0452 . 30442683 PMC6384094

[16] Panda H, Pelakh L, Chuang TD, Luo X, Bukulmez O, Chegini N. Endometrial miR-200c is altered during transformation into cancerous states and targets the expression of ZEBs, VEGFA, FLT1, IKKbeta, KLF9, and FBLN5. Reprod Sci. 2012;19(8):786–96. Epub 2012/05/10. 10.1177/1933719112438448 22569286 PMC4046309

[17] Yeramian A, Santacana M, Sorolla A, Llobet D, Encinas M, Velasco A, et al Nuclear factor-kappaB2/p100 promotes endometrial carcinoma cell survival under hypoxia in a HIF-1alpha independent manner. Lab Invest. 2011;91(6):859–71. Epub 2011/05/04. 10.1038/labinvest.2011.58 21537326

[18] Jiang T, Chen N, Zhao F, Wang XJ, Kong B, Zheng W, et al High levels of Nrf2 determine chemoresistance in type II endometrial cancer. Cancer Res. 2010;70(13):5486–96. Epub 2010/06/10. 10.1158/0008-5472.CAN-10-0713 . 20530669 PMC2896449

[19] Beinse G, Just PA, Rance B, Izac B, Letourneur F, Saidu NEB, et al The NRF2 transcriptional target NQO1 has low mRNA levels in TP53-mutated endometrial carcinomas. PLoS One. 2019;14(3):e0214416 Epub 2019/03/26. 10.1371/journal.pone.0214416 . 30908539 PMC6433262

[20] Girouard J, Lafleur MJ, Parent S, Leblanc V, Asselin E. Involvement of Akt isoforms in chemoresistance of endometrial carcinoma cells. Gynecol Oncol. 2013;128(2):335–43. Epub 2012/11/24. 10.1016/j.ygyno.2012.11.016 . 23174537

[21] Lin H, Zhang M, Yu H, Zhang H, Li Y, Xu J, et al Analysis of differentially expressed genes between endometrial carcinosarcomas and endometrioid endometrial carcinoma by bioinformatics. Arch Gynecol Obstet. 2016;293(5):1073–9. Epub 2015/09/17. 10.1007/s00404-015-3880-1 . 26374646

[22] Karnezis AN, Hoang LN, Coatham M, Ravn S, Almadani N, Tessier-Cloutier B, et al Loss of switch/sucrose non-fermenting complex protein expression is associated with dedifferentiation in endometrial carcinomas. Mod Pathol. 2016;29(3):302–14. Epub 2016/01/09. 10.1038/modpathol.2015.155 . 26743474 PMC4980656

[23] Mendiola M, Redondo A, Heredia-Soto V, Herranz J, Berjon A, Hernandez A, et al Predicting Response to Standard First-line Treatment in High-grade Serous Ovarian Carcinoma by Angiogenesis-related Genes. Anticancer Res. 2018;38(9):5393–400. Epub 2018/09/09. 10.21873/anticanres.12869 . 30194194

[24] Liu CL, Sheu JJ, Lin HP, Jeng YM, Chang CY, Chen CM, et al The overexpression of MYST4 in human solid tumors is associated with increased aggressiveness and decreased overall survival. Int J Clin Exp Pathol. 2019;12(2):431–42. Epub 2020/01/15. . 31933848 PMC6945101

[25] Joshi HP, Subramanian IV, Schnettler EK, Ghosh G, Rupaimoole R, Evans C, et al Dynamin 2 along with microRNA-199a reciprocally regulate hypoxia-inducible factors and ovarian cancer metastasis. Proc Natl Acad Sci U S A. 2014;111(14):5331–6. Epub 2014/04/08. 10.1073/pnas.1317242111 . 24706848 PMC3986124

[26] Papp E, Hallberg D, Konecny GE, Bruhm DC, Adleff V, Noe M, et al Integrated Genomic, Epigenomic, and Expression Analyses of Ovarian Cancer Cell Lines. Cell Rep. 2018;25(9):2617–33. Epub 2018/11/30. 10.1016/j.celrep.2018.10.096 . 30485824 PMC6481945

[27] Hirsch CL, Wrana JL, Dent SYR. KATapulting toward Pluripotency and Cancer. J Mol Biol. 2017;429(13):1958–77. Epub 2016/10/23. 10.1016/j.jmb.2016.09.023 . 27720985 PMC5382138

[28] Baell JB, Leaver DJ, Hermans SJ, Kelly GL, Brennan MS, Downer NL, et al Inhibitors of histone acetyltransferases KAT6A/B induce senescence and arrest tumour growth. Nature. 2018;560(7717):253–7. Epub 2018/08/03. 10.1038/s41586-018-0387-5 . 30069049

[29] Sheikh BN, Lee SC, El-Saafin F, Vanyai HK, Hu Y, Pang SH, et al MOZ regulates B-cell progenitors and, consequently, Moz haploinsufficiency dramatically retards MYC-induced lymphoma development. Blood. 2015;125(12):1910–21. Epub 2015/01/22. 10.1182/blood-2014-08-594655 . 25605372 PMC4440887

[30] Monk BJ, Chapman JA, Johnson GA, Brightman BK, Wilczynski SP, Schell MJ, et al Correlation of C-myc and HER-2/neu amplification and expression with histopathologic variables in uterine corpus cancer. Am J Obstet Gynecol. 1994;171(5):1193–8. Epub 1994/11/01. 10.1016/0002-9378(94)90131-7 . 7977518

[31] Shukla SA, Howitt BE, Wu CJ, Konstantinopoulos PA. Predicted neoantigen load in non-hypermutated endometrial cancers: Correlation with outcome and tumor-specific genomic alterations. Gynecol Oncol Rep. 2017;19:42–5. Epub 2017/01/11. 10.1016/j.gore.2016.12.009 . 28070553 PMC5219603

[32] Buchynska LG, Brieieva OV, Iurchenko NP. Assessment of HER-2/neu, small es, Cyrillic-MYC and CCNE1 gene copy number variations and protein expression in endometrial carcinomas. Exp Oncol. 2019;41(2):138–43. Epub 2019/07/03. 10.32471/exp-oncology.2312-8852.vol-41-no-2.12973 31262163

[33] Onagoruwa OT, Pal G, Ochu C, Ogunwobi OO. Oncogenic Role of PVT1 and Therapeutic Implications. Front Oncol. 2020;10:17 Epub 2020/03/03. 10.3389/fonc.2020.00017 . 32117705 PMC7010636

[34] Rivas S, Gomez-Oro C, Anton IM, Wandosell F. Role of Akt Isoforms Controlling Cancer Stem Cell Survival, Phenotype and Self-Renewal. Biomedicines. 2018;6(1). Epub 2018/03/10. 10.3390/biomedicines6010029 . 29518912 PMC5874686

[35] Takai N, Kawamata N, Walsh CS, Gery S, Desmond JC, Whittaker S, et al Discovery of epigenetically masked tumor suppressor genes in endometrial cancer. Mol Cancer Res. 2005;3(5):261–9. Epub 2005/05/12. 10.1158/1541-7786.MCR-04-0110 . 15886297

[36] Fuchs O. Growth-inhibiting activity of transcription factor C/EBPalpha, its role in haematopoiesis and its tumour suppressor or oncogenic properties in leukaemias. Folia Biol (Praha). 2007;53(3):97–108. Epub 2007/06/21. . 17580000 10.14712/fb2007053030097

[37] Kuhn E, Bahadirli-Talbott A, Shih IeM. Frequent CCNE1 amplification in endometrial intraepithelial carcinoma and uterine serous carcinoma. Mod Pathol. 2014;27(7):1014–9. Epub 2013/12/07. 10.1038/modpathol.2013.209 . 24309323

[38] Cocco E, Lopez S, Black J, Bellone S, Bonazzoli E, Predolini F, et al Dual CCNE1/PIK3CA targeting is synergistic in CCNE1-amplified/PIK3CA-mutated uterine serous carcinomas in vitro and in vivo. Br J Cancer. 2016;115(3):303–11. Epub 2016/06/29. 10.1038/bjc.2016.198 . 27351214 PMC4973158

[39] Leskela S, Perez-Mies B, Rosa-Rosa JM, Cristobal E, Biscuola M, Palacios-Berraquero ML, et al Molecular Basis of Tumor Heterogeneity in Endometrial Carcinosarcoma. Cancers (Basel). 2019;11(7). Epub 2019/07/22. 10.3390/cancers11070964 . 31324031 PMC6678708

[40] Mermel CH, Schumacher SE, Hill B, Meyerson ML, Beroukhim R, Getz G. GISTIC2.0 facilitates sensitive and confident localization of the targets of focal somatic copy-number alteration in human cancers. Genome Biol. 2011;12(4):R41 Epub 2011/04/30. 10.1186/gb-2011-12-4-r41 . 21527027 PMC3218867

[41] Shah SP, Xuan X, DeLeeuw RJ, Khojasteh M, Lam WL, Ng R, et al Integrating copy number polymorphisms into array CGH analysis using a robust HMM. Bioinformatics. 2006;22(14):e431–9. Epub 2006/07/29. 10.1093/bioinformatics/btl238 . 16873504

[42] Olshen AB, Venkatraman ES, Lucito R, Wigler M. Circular binary segmentation for the analysis of array-based DNA copy number data. Biostatistics. 2004;5(4):557–72. Epub 2004/10/12. 10.1093/biostatistics/kxh008 . 15475419

[43] Griffith M, Spies NC, Krysiak K, McMichael JF, Coffman AC, Danos AM, et al CIViC is a community knowledgebase for expert crowdsourcing the clinical interpretation of variants in cancer. Nat Genet. 2017;49(2):170–4. Epub 2017/02/01. 10.1038/ng.3774 . 28138153 PMC5367263

[44] Kusnoor SV, Koonce TY, Levy MA, Lovly CM, Naylor HM, Anderson IA, et al My Cancer Genome: Evaluating an Educational Model to Introduce Patients and Caregivers to Precision Medicine Information. AMIA Jt Summits Transl Sci Proc. 2016;2016:112–21. Epub 2016/08/30. . 27570660 PMC5001739

[45] Chakravarty D, Gao J, Phillips SM, Kundra R, Zhang H, Wang J, et al OncoKB: A Precision Oncology Knowledge Base. JCO Precis Oncol. 2017;2017 Epub 2017/09/12. 10.1200/PO.17.00011 . 28890946 PMC5586540

