# Supplementary figures and images for: KAT6A amplifications are associated with shorter progression-free survival and overall survival in patients with endometrial serous carcinoma
Source: PLoS One. 2020 Sep 2;15(9):e0238477. doi: 10.1371/journal.pone.0238477 (PMC7467277; doi:10.1371/journal.pone.0238477)

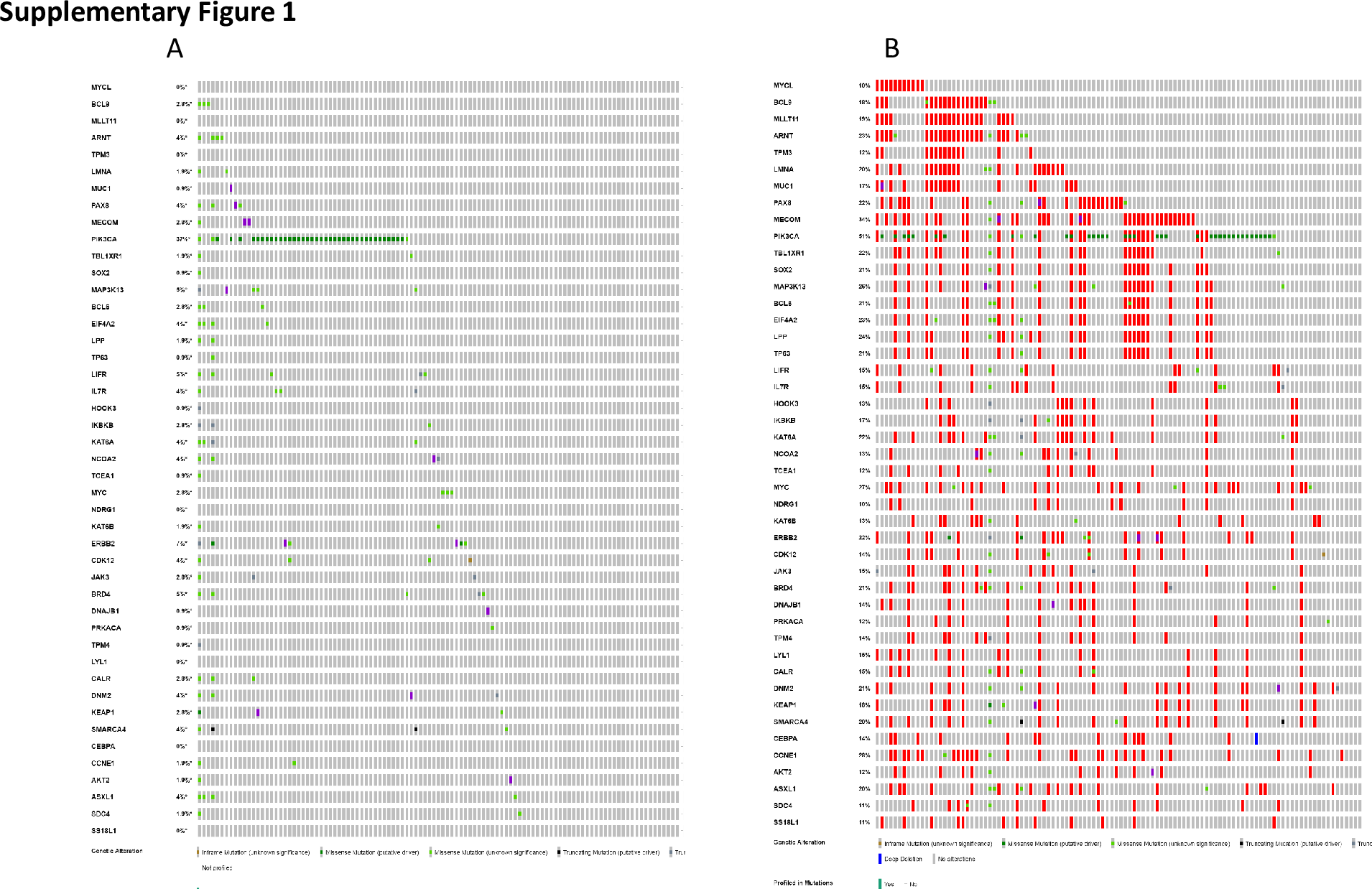

Supplement: S1 Fig — A. Somatic mutations. B. Combined somatic mutations and somatic copy number alterations in 45 Tier 1 CGC-COSMIC genes occurring in the endometrial serous cancers of the TCGA PanCan dataset. (TIF) [file pone.0238477.s005.tif]
